# Zoonotic Potential and Antibiotic Resistance of *Escherichia coli* in Neonatal Calves in Uruguay

**DOI:** 10.1264/jsme2.ME17046

**Published:** 2017-09-27

**Authors:** Ana Umpiérrez, Inés Bado, Martín Oliver, Sofía Acquistapace, Analía Etcheverría, Nora Lía Padola, Rafael Vignoli, Pablo Zunino

**Affiliations:** 1 Departamento de Microbiología, Instituto de Investigaciones Biológicas Clemente Estable Avenida Italia 3318. PC: 11600. Montevideo Uruguay; 2 Departamento de Bacteriología y Virología, Instituto de Higiene, Facultad de Medicina, Universidad de la República Avenida Alfredo Navarro 3051. PC: 11600. Montevideo Uruguay; 3 Laboratorio de Inmunoquímica y Biotecnología, CIVETAN-Facultad de Ciencias Veterinarias, Universidad Nacional del Centro de la Provincia de Buenos Aires General Pinto 399, PC: B7000GHG, Tandil Argentina

**Keywords:** *Escherichia coli*, zoonotic potential, antibiotic multi-resistance, *mcr-1*

## Abstract

*Escherichia coli* is one of the main etiological agents of neonatal calf diarrhea (NCD). The objective of this study was to assess the presence of virulence genes, genetic diversity, and antibiotic resistance mechanisms in *E. coli* associated with NCD in Uruguay. PCR was used to assess the presence of intimin, Shiga-like toxin, and stable and labile enterotoxin genes. Resistance to fluoroquinolones and oxyimino-cephalosporins was estimated on Müller-Hinton agar plates. Further antibiotic disc-diffusion tests were performed to assess bacterial multi-resistance. The presence of PMQR, ESBL, MCR-1, and integron genes was evaluated. Isolates were typed using ERIC-PCR, and 20 were selected for MLST, adhesion to Hep-2 cells, *in vitro* biofilm formation, and eukaryotic cytotoxicity. The prevalence of ETEC genes was lower than 3% in each case (*estA* and *elt*). Six isolates were EPEC (*eae*+) and 2 were EHEC/STEC (*eae*+/*stx1*+). The results of a diversity analysis showed high genetic heterogenicity among isolates. Additionally, different sequence types, including ST10, ST21, and ST69, were assigned to selected isolates. Thirty-six percent (96/264) of the isolates were fluoroquinolone-resistant, with 61/96 (63.5%) being multidrug-resistant. Additionally, 6 were oxyimino-cephalosporin-resistant. The *qnrB*, *qnrS1*, and *bla*_CTX-M-14_ genes were detected, whereas no isolates carried the *mcr-1* gene. Isolates had the ability to adhere to Hep-2 cells and form biofilms. Only 1 isolate expressed toxins *in vitro*. *E. coli* from NCD cases in Uruguay are very diverse, potentially virulent, and may interact with eukaryotic cells. Zoonotic potential, together with resistance traits and the presence of horizontal transfer mechanisms, may play a significant role in infections caused by these microorganisms.

Diarrheagenic *Escherichia coli* infections have been a health concern for decades. *E. coli* is one of the main etiological agents responsible for neonatal calf diarrhea (NCD), and represents an economic challenge in livestock and dairy industries worldwide (33). The severity of NCD infections may be associated with a single or various risk factors acting simultaneously; however intensive animal breeding systems have recently increased the transmission of infectious diseases such as NCD (33). Enterotoxigenic (ETEC) and enteropathogenic *E. coli* (EPEC) are two of the most common pathotypes associated with NCD, and are responsible for high morbidity and mortality rates (27, 36). Additionally, enterohemorrhagic (EHEC)/Shiga toxin producer *E. coli* (STEC) are relevant because they are important zoonotic pathogens, and bovines have been identified as their natural reservoir (19, 39).

*E. coli* toxins are worldwide-distributed virulence factors involved in NCD and human infections. ETEC heat-labile enterotoxin (L-T) and heat-stable enterotoxin (S-T) are responsible for the hypersecretion of fluids into the intestinal lumen, triggering dehydration and acidosis in neonatal calves (35, 41). Stx toxins are regarded as the most important virulence factor of STEC because they induce the apoptosis of intestinal cells and are associated with human hemolytic uremic syndrome (HUS) and hemorrhagic colitis (HC) outbreaks every year (18).

The intestinal microbiota, as well as pathogenic microorganisms, intimately interact with the eukaryotic host. This interaction depends on the microorganism and host, as well as the stage of infection in the case of pathogens. The ability of pathogenic *E. coli* to adhere to epithelial cells and persist in biofilms has important implications in the early stages of bacterial infection (27). Additionally, the expression of toxins is a crucial step in the infection process. The toxicity of Shiga toxins 1 and 2 toxins of EHEC/STEC have been the focus of numerous studies, mainly because of the lethal effects of human infections (54).

Antibiotic therapy is frequently used to treat different infectious diseases in animals, including NCD. While this therapy may be associated with a mortality rate reduction in some cases, the indiscriminate use of antibiotics has been accompanied by an increase in bacterial resistance, generating important public health issues and economic losses in production industries in recent years (10). The most commonly used antibiotics in animals include β-lactams, aminoglycosides, fluoroquinolones, and tetracyclines (15). The first 3 groups are also widely used in the treatment of various infectious diseases in humans; therefore, selection pressure exerted by an inadequate use may have a direct impact on public health.

The transference of resistance genes has become relevant in recent years due to cross-contamination between food-production chains, animals, and humans (38). There are currently no studies available on antimicrobial resistance genes in bovine *E. coli* in Uruguay. However, the presence of class 1 integrons, extended-spectrum β-lactamases (ESBL), class C β-lactamases (AmpC), and plasmid-mediated quinolones resistance genes (PMQRg) has been detected in *Salmonella enterica* and/or *E. coli* in our country (3, 4, 16, 58, 59). Among transferable resistance genes, plasmid colistin resistance genes (*mcr-1* and *mcr-2*), which have already been identified in humans, but mainly in livestock and farm animals, have emerged around the world as a new threat to human health (32).

The aim of this study was to detect the presence and distribution of genes that allow the identification of EHEC/STEC and ETEC in bovines in order to elucidate the *E. coli* population structure, assess antibiotic resistance, and evaluate interactions between bacteria and eukaryotic cells. Additionally, we performed the first survey in our country on plasmid genes involved in extended-spectrum cephalosporins, quinolones, and colistin resistance in *E. coli* isolated from bovines.

## Methods

### E. coli isolates

A total of 303 *E. coli* isolates obtained from different animal facilities throughout Uruguay were analyzed in this study: 297 were collected from calves younger than 35 d old between 2012 and 2013 (55), and 6 were from 6-month-old symptomatic calves in 2014 (this study). All isolates were biochemically identified and 16S RNA genes were sequenced for identity confirmation. A total of 242 isolates from animals with symptoms and 61 isolates from healthy animals were analyzed throughout this study.

### Bacteriological culture and growth conditions

Isolates were routinely grown on Tryptone Soy Agar plates (TSA) or Tryptone Soy Broth (TSB) (Oxoid) at 37°C for 18–24 h. In antibiotic tests, isolates were cultured in Müller-Hinton agar (Oxoid) at 37°C for 24 h.

### Genomic DNA extraction

Genomic DNA was extracted using the GenElute Bacterial Genomic DNA kit (Sigma). DNA quantification was performed using NanoDrop (Thermo Scientific).

### Identification of virulence genes

The presence of 5 *E. coli* virulence genes was evaluated by conventional PCR. Primers for the partial amplification of *stx1* and *stx2*, *eae*, and *estA* and *elt* (coding Shiga-like toxins 1 and 2, intimin, and S-T and L-T enterotoxins, respectively) were used as previously described (22, 42, 44).

### ERIC-PCR

The diversity of *E. coli* isolates was analyzed by ERIC-PCR as described by Versalovic (57). Gel images were analyzed by GelCompar II software (Applied Maths, Version 6.5). Dendrograms were constructed using the UPGMA method and Dice coefficient. Isolates were grouped into the same genetic variant when similarity values were ≥75% and were considered to be the same strain when similarity values were ≥98% (11). Genetic variants were used to assess the Shannon Diversity Index (*H′* ) of the collection (8). The *H′* index was calculated according to the following equation:

$$H^{\prime }=-\sum_{i=1}^{S}p_{i}\;\text{ln}\;p_{i}$$

where *S* is number of unique genetic variants, *pi* is number of isolates sharing the same genetic variant (*i*)/total number of isolates.

*E. coli* isolates were separated into different groups according to ERIC-PCR patterns (genetic variants), virotyping (presence of virulence genes), collection dates, and animal symptoms. All non-duplicate isolates were selected for later susceptibility studies. Twenty of these isolates were selected as representatives of the different isolates groups and were used for multilocus sequence typing (MLST), adhesion to Hep-2 cells, *in vitro* biofilm formation, and eukaryotic cytotoxicity. Additionally, MLST was performed on 6 isolates that harbored PMQR and ESBL resistance genes.

### MLST

MLST was performed on the selected *E. coli* isolates following the method described by Wirth *et al.* (61). Seven housekeeping genes (*adk*, *fumC*, *gyrB*, *icd*, *mdh*, *purA*, and *recA*) were partially amplified by PCR using previously described primers (61). Allele numbers and sequence types (ST) were assigned in accordance with the *E. coli* MLST database (http://mlst.warwick.ac.uk/mlst/). The concatenated gene sequence of each isolate was used to generate phylogenetic trees with MEGA software (Version 6.06), the Neighbor-Joining method, and 1,000 bootstrap replicates.

### Antimicrobial susceptibility analyses

In order to detect isolates with some degree of resistance to fluoroquinolones and oxyimino-cephalosporins, we performed 2 screens with ciprofloxacin (CIP) and cefotaxime (CTX). CIP screening was performed according to the proposed ECOFF value to this antibiotic by EUCAST (http://www.eucast.org) and as recommended by Cavaco *et al.* (10). Briefly, bacterial suspensions were adjusted to 0.5 Mc Farland and plated onto Müller Hinton agar plates supplemented with CIP (0.125 μg mL^−1^) (MH-CIP) at 37°C for 24 h. CTX screening was performed using the same protocol at a concentration of CTX of 1 μg mL^−1^ (MH-CTX). *E. coli* ATCC 25922 was used as a quality control. Each isolate was cultured in duplicate. Isolates that grew were considered for later studies.

Isolates grown in MH-CIP and MH-CTX were analyzed using the Kirby-Bauer disc-diffusion method, according to the Clinical Laboratory Standard Institute (CLSI) (45). Fourteen different antibiotics were examined: amoxicillin-clavulanic acid (AMC), ampicillin (AMP), ceftriaxone (CRO), ceftazidime (CAZ), cefepime (FEP), imipenem (IMP), meropenem (MEM), nalidixic acid (NA), CIP, enrofloxacin (ENR), gentamicin (CN), amikacin (AK), trimethoprim-sulfamethoxazole (SXT), and fosfomycin (FOS). All antibiotic discs were from Oxoid. Quality control was performed with *E. coli* ATCC 25922 and ATCC 35218. The interpretation of results was performed according to CLSI 2016, except for ENR, which was interpreted using Veterinary Antimicrobial Susceptibility Testing (VAST) (60). The presence of ESBL and AmpC was evaluated by the synergy double-disc method in isolates that were resistant to 3rd generation cephalosporins using ESBL and AmpC inhibitors (AMC and boronic acid) plus a 3rd generation cephalosporin according to Cordeiro *et al.* (16) and the European Committee on Antimicrobial Susceptibility Testing (EUCAST) (www.eucast.org). All non-duplicate *E. coli*, as previously described, including susceptibility results obtained so far, were selected for further studies (*n*=264).

Antibiotic resistance patterns were generated using AMP (A), oxyimino-cephalosporins (O), CN (G), SXT (S), fluoroquinolones (Q), and FOS (F). Oxyimino-cephalosporin resistance was considered if resistance to CRO and/or CAZ and/or FEP was observed. Fluoroquinolone resistance (Q) was considered if resistance to CIP and/or ENR was observed. Isolates were considered to be multiresistant if non-susceptibility to at least one agent on three or more antimicrobial categories appeared (16).

All isolates that showed CIP disk diffusion inhibition zones ≤20 mm were considered to be resistant, whereas those that displayed a zone diameter of 21–30 mm were considered with a low level of CIP resistance screening (LLCR) (1).

### Identification of PMQR and ESBL genes

The presence of PMQR and ESBL genes was evaluated by PCR. The partial amplification of *qnrA*, *qnrB*, *qnrC*, *qnrD*, *qnrS*, and *qepA* genes was performed following previously described protocols (10, 28). The presence of *bla*_SHV_, *bla*_PER-2_, *bla*_TEM_, and *bla*_CTX-M_ genes was evaluated in isolates that were positive in the synergy double-disc technique accordingly to our previous studies (3, 58). Plasmid-encoded AmpC (pAmpC) alleles were detected by multiplex PCR according to Pérez-Pérez *et al.* (46). All PCR products were confirmed by direct sequencing.

### Presence of integrons

Class 1 and class 2 integron genes were studied in selected *E. coli* isolates. The amplification of *int1*, *int2*, *qacEΔ1*, and *sul1* genes was performed according to previous studies (34, 58). The variable region of the class 1 integron was sequenced in isolates possessing *int1* (5′ integron region) and *qacEΔ1* and *sul1* (3′ integron region) genes using the 5CS and 3CS primers, as previously described (3, 58).

### Colistin resistance mechanism

The colistin resistance gene *mcr-1* was detected according to Liu *et al.* (32) in the whole collection.

### Adhesion to Hep-2 culture cells

The ability of *E. coli* isolates to adhere to Hep-2 cells was evaluated using a previously reported protocol by Etcheverría *et al.* (21). Briefly, confluent Hep-2 monolayers were inoculated with bacterial suspensions (0.5 Mc Farland scale). Each isolate was inoculated in duplicate. After 3 h, monolayers were washed, and cells were detached with Trypsin-EDTA. The percentage of adhesion (adhered and internalized bacteria) with respect to total cell numbers was enumerated by colony counts on TSA plates after a 24-h incubation at 37°C.

### Cytotoxicity assay

The cytotoxicity of *E. coli* isolates was evaluated in Vero cells monolayers (30). Bacterial supernatants were inoculated onto Vero monolayer wells (×2) for 48 h. Eukaryote cells were then stained with 10% methylene blue and fixed with methanol. The interpretation of monolayer destruction was separately evaluated by two different scientists in a blind mode. *E. coli* O157:H7 supernatant was used as a positive control (2).

### Biofilm formation

*In vitro* biofilm formation was evaluated according to the protocol described by Pratt and Kolter (48). *E. coli* isolates were cultured in TSB wells (×3) at 37°C for 48 h. *Proteus mirabilis* 2921 was used as a positive control (65). Biofilms were dyed with crystal violet (CV) and then solubilized with ethanol. The absorbance of each well was measured at 590 nm and the interpretation of results was performed using the Angel-Villegas criteria (2).

## Results

### Presence of virulence genes

After PCR analyses, *eae* showed a prevalence of 2.6% (8/303), corresponding to the highest prevalence of all evaluated genes (Table 1). Two isolates (2/303) were classified as EHEC/STEC (*stx1*+/*eae*+), whereas 6 *eae*+ isolates (6/303) were classified as EPEC. The prevalence of *elt* and e*stA* was 2.6% (8/303) in both analyzed genes (Table 1).

### *E. coli* genomic diversity

Genetic differences between *E. coli* isolates were observed after ERIC-PCR. However, a clear pattern was not obtained within the entire collection. ERIC-PCR analyses showed high genetic heterogenicity within the *E. coli* collection (Fig. S1), which was then confirmed with the Shannon Diversity Index, *H′*=2.89. As exemplified in Fig. S1, *E. coli* isolates obtained in close herds (adjacent herd facilities) were grouped in the same variants. We observed different genetic variants in the same bovine herd. Certain genetic variants were even found in healthy or symptomatic calves (Fig. S1).

### MLST analysis

**Fig. 1:**
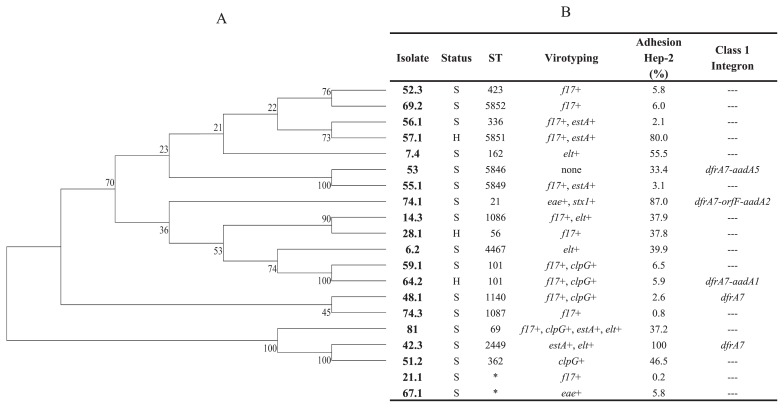
Assigned ST, percentage of adhesion to Hep-2 monolayers, and presence of class 1 integrons. (A), *E. coli* phylogeny of 7 concatenated housekeeping gene sequences using the Neighbor-Joining method. Bootstrap values are a product of 1,000 replicates. (B), Assigned ST, adhesion percentages to the Hep-2 monolayers of bovine *E. coli* isolates, and the presence of Class 1 integrons. S, animal with symptoms; H, healthy animal. Virotyping: *f17*, fimbriae 17 gene; *clpG*, CS31A adhesion gene; *f5*, fimbriae F5 gene. The presence of F17, F5, and CS31A genes were previously evaluated (55). *, ST not assigned.

MLST was performed on selected isolates with the objective of establishing the populational structure of our *E. coli* collection. Isolate selection criteria were based on virotyping, the geographical origin of the isolates, and ERIC-PCR grouping, as previously described. Seventeen different ST were found, including 4 novel ST (Fig. 1B). The only repeated ST was ST101, which was assigned to 2 *E. coli* isolates from a single farm. These isolates were obtained from an animal with diarrhea, whereas the other animal had no symptoms. Novel ST were submitted to the Warwick MLST database and classified as ST5846 (isolate 53), ST5849 (isolate 55.1), ST5851 (isolate 57.1), and ST5852 (isolate 69.2) (Fig. 1B). It was not possible to assign 2 *E. coli* isolates (21.1 and 67.1) to any ST because a new *adk* gene allele must be identified in both isolates.

After comparing our results with the Warwick MLST database, isolates assigned to previously described ST grouped with the pathogen *E. coli* strains (ExPEC, EHEC/STEC, APEC, and EAEC) of animal and human origins. ST69 was assigned to 1 bovine *E. coli* isolate (8.1), whereas our *eae*+/*stx1*+ isolate (74.1) was assigned to ST21, a member of the ST29 complex.

Phylogenetic analyses on concatenated housekeeping gene sequences showed previous genetic heterogenicity observed with ERIC-PCR. Isolates assigned to ST101 grouped together (isolates 59.1 and 64.2), whereas those assigned to new ST and different geographical origins (isolates 53 and 55.1) also grouped together (Fig. 1A).

### Antimicrobial susceptibility analyses

According to previously established criteria, we analyzed 264/303 non-redundant *E. coli* isolates. A total of 112/264 *E. coli* isolates (42.4%) grew on Müller-Hinton agar plates supplemented with 0.125 μg mL^−1^ of CIP and were considered to have reduced sensitivity to this antibiotic. Additionally, 6/264 (2%) grew on Müller-Hinton agar plates supplemented with 1.0 μg mL^−1^ of CTX, including 3 with resistance to CIP and CTX.

Hence, the disc-diffusion test was used to evaluate 115 *E. coli* isolates. Of these, 84 were resistant to CIP (zone diameter less than or equal to 20 mm), whereas 12 isolates showed LLCR. When fluoroquinolone-resistant isolates were considered (resistance to CIP and/or ENR), 92 were fluoroquinolone-resistant. Additionally, 4 isolates showed LLCR.

Considering CTX resistance, 3 out of 6 isolates resistant to oxyimino-cephalosporins were also resistant to fluoroquinolones.

A total of 99 isolates were analyzed, including: 84 isolates resistant to CIP (3/84 were ESBL-positive), 12 LLCR, and 3 isolates that were resistant to oxyimino-cephalosporins, but susceptible to CIP. All were distributed in 18 different susceptibility profiles and 75/99 (75.8%) were concentrated in 4 profiles (Table 2).

Sixty-one out of 99 isolates (61.6%) showed multidrug resistance; explaining the profiles QAS and QASG 54/61 (82%) of multi-resistant isolates (profiles 1 and 2, Table 2). Three out of 59 MDR isolates were also resistant to FOS (profiles 10 to 12, Table 2).

Three isolates were resistant to fluoroquinolones and oxyimino-cephalosporins and displayed a positive synergy test between CRO, CAZ, and AMC (profile 8, Table 2). Another 3 isolates showed susceptibility to CIP, but resistance to oxyimino-cephalosporins and a positive synergy test between CAZ, CRO, and boronic acid (profile 7, Table 2). A total of 264 isolates were susceptible to AK and carbapenems.

### Presence of PMQR, ESBL, and transferable colistin resistance gene (mcr-1)

PMQR genes were evaluated in 96 *E. coli* isolates (84 FQ-resistant and 12 LLCR). PMQR genes were detected in 7/96 isolates: 6/96 were *qnrB2*+ (isolates 4.1, 4.4, 7.4, 9.1, 9.2, and 73.2) and 1/96 was *qnrS1*+ (isolate 68.1). All positive isolates were obtained from symptomatic animals, and 7.4, 9.1, and 9.2 also showed multidrug resistance to antibiotics. Three *E. coli* strains displayed a double disc synergy test with clavulanic acid and were *bla*_CTX-M-14_ producers (21.3, 22.1A and 22.1B). On the other hand, 3 isolates were positive for the boronic acid test; however, we were unable to confirm any plasmidic AmpC β-lactamase gene (Table 2).

Transferable colistin resistance gene (*mcr-1*) was not detected in any *E. coli* isolate.

### Integron-mediated resistance

The presence of class 1 integrons was examined in 5/20 evaluated *E. coli* isolates (7.4, 42.3, 48.1, 53, and 64.2). All 5 isolates were positive for *int1*, *qacEΔ1*, and *sul1* by PCR. Integron sequencing revealed 4 different gene arrangements: *dfrA7*; *dfrA17-aadA5*; *dfrA1-aadA1*; *dfrA12-orfF-aadA2* (Fig. 1B). No class 2 integron genes were detected.

### MLST distribution of qnr/ESBL producers

**Fig. 2:**
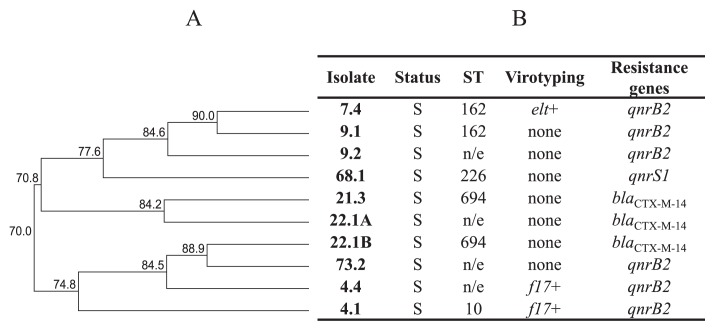
ST distribution of *qnr* and ESBL *E. coli* producers. (A), Dendrogram generated with the UPGMA method (GelCompar II). Ten *E. coli* isolates that showed resistance genes. (B), Assigned ST and resistance genes of bovine *E. coli* isolates. S, animal with symptoms. H, healthy animal. Virotyping: *elt*, LT gene (this work); *f17*, fimbriae 17 gene (previously evaluated [55]). n/e, not evaluated.

Four different ST were detected within *qnr*+ isolates or ESBL producers: ST10, ST162, ST226, and ST694 (Fig. 2), with two being repeated. ST162 was assigned to 2 *qnr*+ *E. coli* isolates, while ST694 was assigned to 2 *bla*_CTX-M-14_ *E. coli* isolates (Fig. 2B).

### Hep-2 adhesion assay

The colony plate counts of adhered and internalized bacteria to Hep-2 cells showed variable adhesion percentages within *E. coli* isolates. As shown in Fig. 1B, adhesion was calculated as the mean of colony counts in two different Hep-2 wells. Nine *E. coli* isolates (45%) had an adhesion value that was lower than 10%, whereas 11 isolates showed adhesion values that were greater than 30% (Fig. 1B.). Within the last group, only 3 isolates had an adhesion value that was higher than 80% (isolates 42.3, 57.1, and 74.1) (Fig. 1B).

### Cytotoxic effect

The expression of *E. coli* cytotoxins was evaluated in Vero culture cells, as described in the Materials and methods. The destruction of Vero monolayers was analyzed by light microscopy and the interpretation of results was performed by two observers in a blind mode. Only 1/20 of the evaluated *E. coli* isolates showed a destructive phenotype. *E. coli* 74.1 isolate, which was previously classified as an EHEC/STEC by PCR (*eae*+/*stx1*+), showed a toxic phenotype, destroying more than 90% of Vero monolayer wells with respect to non-treated cells. This percentage of damage was even greater than that produced by positive control *E. coli* O157: H7 (80%) (Fig. S2).

### *In vitro* biofilm formation

The same 20 *E. coli* isolates selected were further analyzed for *in vitro* biofilm formation. All isolates were cultured in triplicate and values are presented as their mean (Fig. S3). The interpretation of results was performed using the Angel-Villegas criteria, as previously described in the Materials and methods. The *in vitro* ability to form biofilms varied amongst isolates. Ten isolates (50%) showed no or a weak ability to form biofilms *in vitro* (isolates 8.1, 14.3, 21.1, 48.1, 55.1, 57.1, 59.1, 64.2, 67.1, and 74.3). On the other hand, only 1 isolate had a moderate ability to form biofilms (isolate 36.4), and 45% of *E. coli* isolates showed a strong ability to form biofilms (isolates 6.2, 7.4, 28.1, 42.3, 51.2, 52.3, 53, 56.1, and 74.1) (Fig. S3).

## Discussion

In the present study, the prevalence of ETEC genes (*estA* and *elt*) was lower than 3% in each case, which was lower than previous findings in the region (47). Within the 303 isolates examined, 6 belonged to EPEC (*eae*+) and only 2 to EHEC/STEC (*eae*+/*stx1*+). No isolate was *stx2*+. The low prevalence of these genes differed from the findings obtained in Argentina and Brazil, both geographically very close to our territory, and revealed molecular *E. coli* differences between bordering countries (7, 53). In support of these results, unlike endemic HUS and HC in Argentina, these infections are a sporadic health concern in Uruguay nowadays (56).

Additionally, diversity analyses established a high genetic heterogenicity among *E. coli* isolates of bovine origin, highlighting the complexity of the NCD scenario. We found different genetic variants in the same bovine herd. Even certain genetic variants were detected in healthy or symptomatic calves. Furthermore, given the low prevalence of ETEC, EPEC, and EHEC/STEC virulence genes, no relationships were detected with these genes and symptoms between isolates. Hence, other factors such as adequate nutrition and colostrum consumption, animal hygiene, or co-infection with other pathogens are risk factors that may contribute to the onset of the disease (33, 37).

Within MLST analyses, 17 different ST were found in the 20 evaluated isolates, with 4 being assigned to new ST (ST5846, ST5849, ST5851, and ST5852). Only 1 out of 17 ST was repeated (ST101). Similar results, including *E. coli* strains of human and bovine origins, established the occurrence of a high number of different ST circulating in Argentina (9). Some previously reported ST have been assigned exclusively to *E. coli* of animal origin, whereas most have been assigned to *E. coli* isolates of animal and human origins. Of these, ST69 has been widely characterized in ExPEC infections (particularly in human UPEC infection) (52), including human infections in our country (59); however, it has also been detected in environmental samples, such as freshwater (40). On the other hand, one *E. coli* isolate was affiliated to ST21, a member of the ST29 complex. ST29 emerged in the mid-1990s in Germany as an *E. coli* O26:H11/H-human clone (64), and has also more recently been detected in healthy animals in Switzerland and included new virulent clones of EHEC O26: H11/H- and STEC O26 in Europe (6, 12, 24, 66). Therefore, *E. coli* isolates assigned to ST that include highly virulent human pathogens highlights the zoonotic role of bovine *E. coli* in Uruguay.

Regarding *qnr*+ or ESBL producer isolates, 3 out of 4 detected ST (ST10, ST162, and ST226) have previously been associated with human infection (5, 13, 14). Two were repeated: ST162 (2 *qnr*+ *E. coli* isolates) and ST694 (2 *bla*_CTX-M-14_ *E. coli* isolates). This finding highlights the role of bovine cattle as a reservoir of human pathogens carrying diverse resistance mechanisms.

Antibiotic resistance has been recognized for decades as a major health issue, and, more recently, a call for attention regarding the emergence of multi-resistance has led to debates on the more frequent occurrence of non-effective treatments for infectious diseases (38). Antibiotic resistance to 14 antibiotics was tested using Kirby-Bauer’s disc-diffusion method according to the CLSI. Fluoroquinolone resistance was detected in 36.4% (96/264) of isolates. This is an important concern, taking into account the frequent use of ENR to treat different infections in animals, particularly poultry. Twelve out of 96 isolates showed LLCR, and only one carried the *qnrB2* gene. The occurrence of LLCR isolates may be due to the presence of PMQR genes or mutations in *gyrA* and/or *gyrB* genes, which confer resistance, but do not reach the breakpoint selected in this study. This may be a cause for concern because isolates with susceptibility to CIP between 21–30 mm may be regarded as susceptible using CLSI breakpoint criteria. Rodriguez-Martinez *et al.* (49) suggested the use of an epidemiological cut-off for CIP of EUCAST (0.032 mg L^−1^) in *E. coli* isolates for the detection of LLCR, which is more suitable to predict resistance *in vivo*.

Studies in the USA, Spain, and the Netherlands reported that fluoroquinolone-based therapy such as ENR and sara-floxacin in birds is responsible for the occurrence of resistant *Campylobacter* spp. in human infections (20, 43, 50). Although ENR is used in Uruguay, there are no records of its administration frequency or doses. Concerning oxyimino-cephalosporin resistance, only 3 (21.3, 22.1A, and 22.1B) out of 96 isolates showed an ESBL phenotype, corresponding to ESBL CTXM-14. CTX-M-14 and CTX-M-15 are considered to be the most prevalent members of the ESBL CTX-M group within multi-resistant *E. coli* (51). The most frequently identified ESBLs in Uruguay are CTX-M-15, CTX-M-14, CTX-M-2, SHV-5, and SHV-12 among others, and have only been detected in human clinical samples to date (3, 58, 59). Besides multi-resistant *E. coli* phenotypes, genetic analyses on transferable resistant mechanisms are imperative. In this study, we detected the presence of *qnrB* and *qnrS1* PMQR genes in *E. coli* isolates of bovine origin. The prevalence of both genes was low, but similar to those found in human and animal samples in Argentina and other countries (1, 17, 25, 29, 62). This is the first study to report on the *qnrS1* gene in Uruguay.

Transferable polymyxin resistance has very recently become a focus of attention (32). The emergence of MCR-1 has alarmed academic and clinical fields because polymixin is regarded as one of the last effective antibiotic groups against intra-hospital Gram-negative infections. We herein report the absence of the *mcr-1* gene in our collection of *E. coli* isolates of bovine origin.

The presence of a class 1 integron was detected in 5 of the *E. coli* isolates analyzed. Four different variable regions were identified (*dfrA7*, *dfrA17-aadA5*, *dfrA1-aadA1*, and *dfrA12-orfF-aadA2*), which may confer resistance to aminoglycosides and trimethoprim to our *E. coli* isolates. The gene cassettes *dfrA17-aadA5* and *dfrA12-orfF-aadA2* have been reported in calves and poultry strains (26), whereas *dfrA17-aadA5* has been detected in *E. coli* strains isolated from adults and children hospitalized in Uruguay (3, 23, 31, 63).

The ability of pathogenic microorganisms to adhere to the intestinal epithelium is essential for the enteric infectious processes of bacteria such as *E. coli*. Adhesion to Hep-2 cells and *in vitro* biofilm formation were detected. However, no relationships were observed among these properties and virotyping (virulence genes), genetic diversity (ERIC-PCR), and clonal distribution (MLST). Only 1 isolate (74.1) had the ability to damage Vero cells, confirming the *in vitro* expression of the Stx toxin in this isolate. Furthermore, the absence of Vero monolayer disruption with the other isolates evaluated demonstrated the lack of other cytotoxic factors, different from Stx.

Our results support *E. coli* associated with NCD being a genetically heterogeneous group that carry virulence-related genes and interact with eukaryotic cells *in vitro*. Zoonotic potentiality, as revealed by MLST, combined with resistance traits and the presence of horizontal gene transfer mechanisms may play a significant role in human infections caused by these microorganisms, favoring inter-species resistance transmission and hindering methods to treat bacterial infections.

## Supplementary Material



## Figures and Tables

**Table 1:** Prevalence of *E. coli* virulence genes

	Symptoms *n_S_*=242	Healthy *n_H_*=61	Total_isolates_ *n*=303
*eae*	5 (2.1%)	3 (4.9%)	8 (2.6%)
*stx1*	2 (4.8%)	0 (0%)	2 (0.7%)
*stx2*	0 (0%)	0 (0%)	0 (0%)
*estA*	6 (2.5%)	2 (3.3%)	8 (2.6%)
*elt*	6 (2.5%)	2 (3.3%)	8 (2.6%)

*n_S_*, number of *E. coli* isolates recovered from animals with symptoms of diarrhea; *n_H_*, number of *E. coli* isolates recovered from healthy animals. Brackets: prevalence of each gene.

**Table 2:** Antibiotic resistance profiles and presence of ESBL/PMQR genes

Profile	Antibiotic resistance profile	No. of isolates	ESBL	PMQR
1	AMP, FQ, SXT	36	ND	—
2	AMP, FQ, CN, SXT	14	ND	*qnrB2* (1)
3	AMP, FQ	13	ND	—
4	FQ	12	ND	*qnrS1* (1)
5	FQ, SXT	4	ND	*qnrB2* (2)
6	AMP, CXM, FQ, SXT	4	ND	—
7	AMP, AMC, CXM, CTX, CAZ	3	ND	—
8	AMP, AMC, CXM, CTX, CAZ, FEP, FQ	3	*bla*_CTX-M-14_ (3)	—
9	AMP, FQ, CN	1	ND	—
10	AMP, FQ, SXT, FOS	1	ND	—
11	AMP, FQ, CN, SXT, FOS	1	ND	—
12	AMP, CXM, FQ, SX, FOS	1	ND	—
13	AMP, CXM, FQ, CN, SXT	1	ND	*qnrB2* (1)
14	AMP, CIP^*^	1	ND	—
15	AMP, CIP^*^, CN, SXT	1	ND	*qnrB2* (1)
16	AMP, CIP^*^, SXT	1	ND	—
17	FQ, CN, SXT	1	ND	*qnrB2* (1)
18	Nal, CIP*	1	ND	—

Total		99	3	7

AMP, Ampicillin; FQ, fluoroquinolones; SXT, trimethoprim-sulfamethoxazole; CN, gentamicin; CXM, cefuroxime; CTX, cefotaxime; CAZ, ceftazidime; FEP, cefepime; AMC, amoxicillin-clavulanic acid; FOS, fosfomycin; CIP*, Low-level ciprofloxacin resistance; Nal, Nalidixic acid.

Brackets: number of isolates showing ESBL (*bla*+) or PMQR (*qnr*+) genes within the antibiotic resistance profile.

ND: Not done.
