# Supplementary material for: Zoonotic Potential and Antibiotic Resistance of Escherichia coli in Neonatal Calves in Uruguay
Source: Microbes Environ. 2017 Sep 27;32(3):275–82. doi: 10.1264/jsme2.ME17046 (PMC5606698; doi:10.1264/jsme2.ME17046)
Supplement: Supplementary file 1 [file 32_275_s1.pdf]

Fig. S1. ERIC-PCR diversity

Exemplifying dendogram generated with UPGMA method (GelCompar II). 49 *E. coli* isolates where obtained from 3 close herds. ★: animals with symptoms. ●: healthy animals.

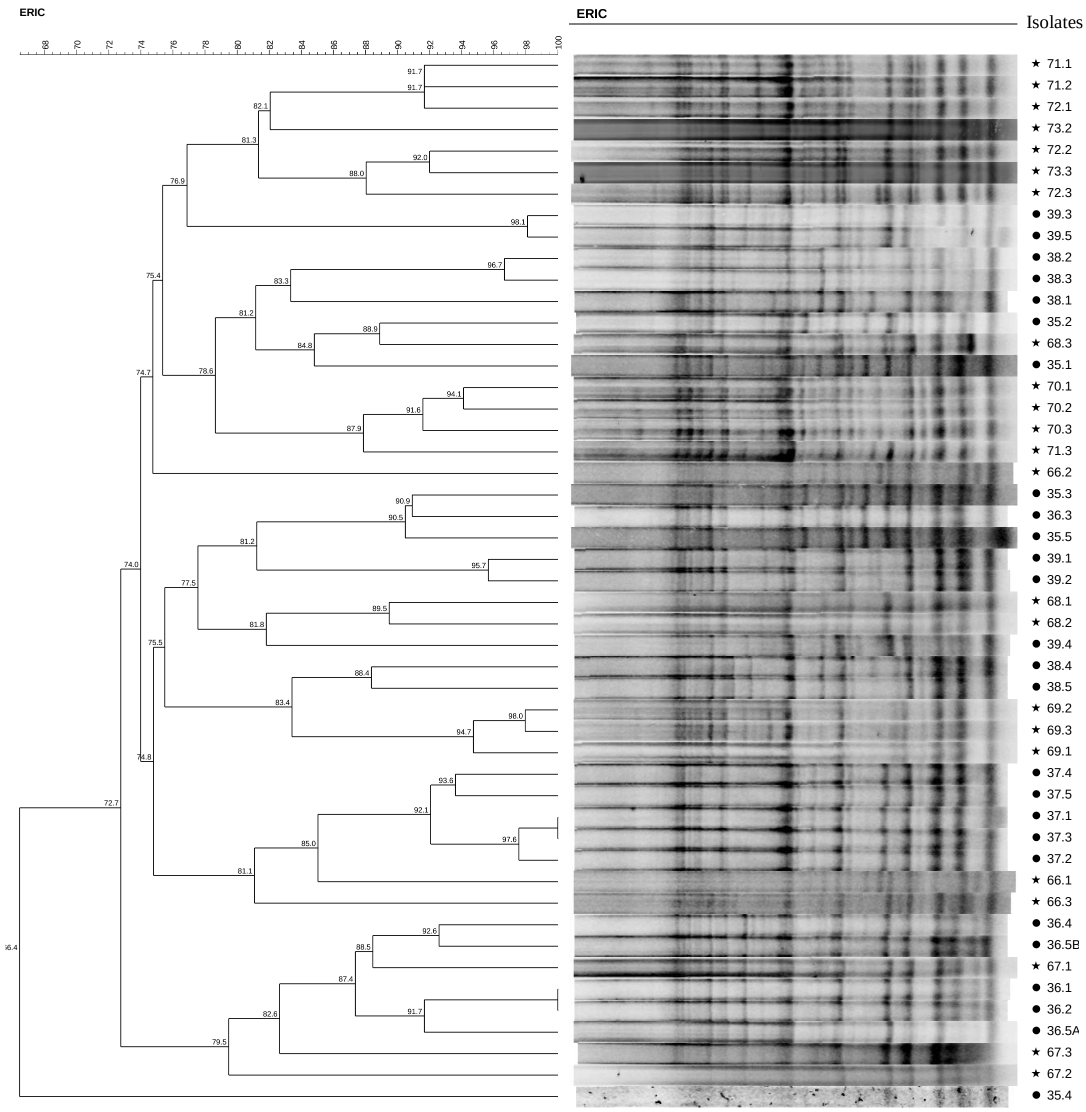

Fig. S2. Cytotoxic assay

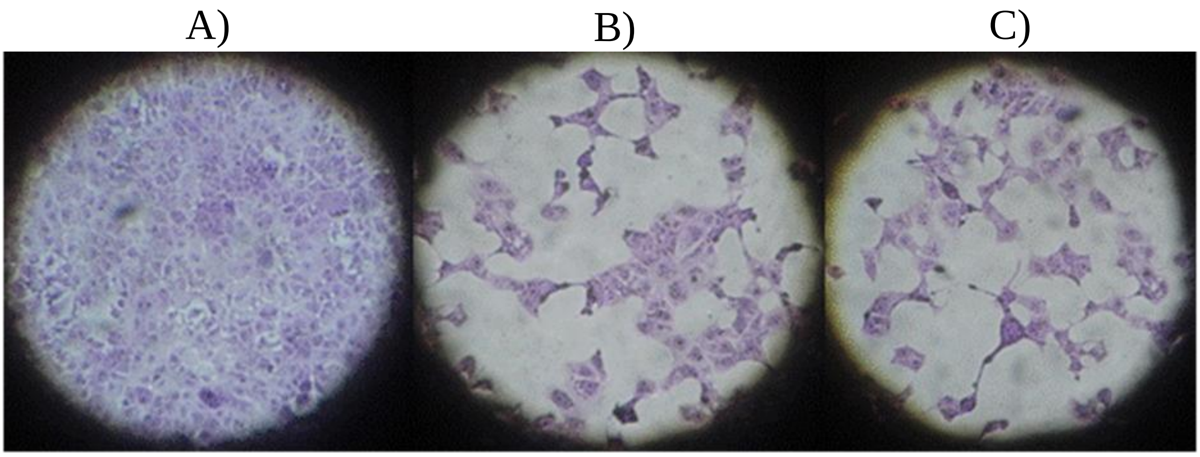

(A) intact Vero monolayer (40x), (B) Vero cells treated with *E. coli* O:157H7, 80% of monolayer destruction (60x), (C)74.1 *E. coli* isolate (*stx1*+, *eae*+), 90% of monolayer destruction (60x).

Fig. S3. In vitro biofilm quantification

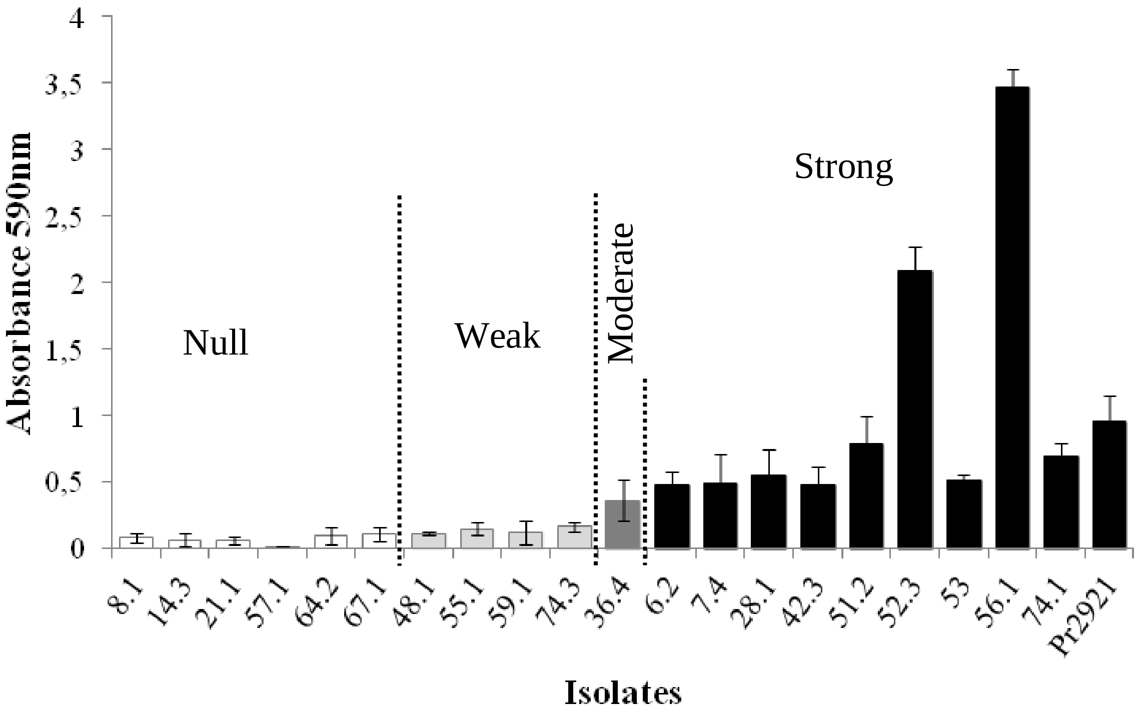

Mean of absorbance's values of each *E. coli* isolate evaluated by triplicate. White and light grey: null and weak biofilm producers. Grey: moderate biofilm producers. Black: strong biofilm producers. Positive control: *P. mirabilis* 2921.
